# Identification of gram-negative bacteria from critical control points of raw and pasteurized cow milk consumed at Gondar town and its suburbs, Ethiopia

**DOI:** 10.1186/1471-2458-12-950

**Published:** 2012-11-06

**Authors:** Legesse Garedew, Ayalew Berhanu, Desalegne Mengesha, Getachew Tsegay

**Affiliations:** 1 Faculty of Veterinary Medicine, University of Gondar, P. O. Box 196, Gondar, Ethiopia

**Keywords:** Critical control points, Gram-negative staining bacteria, Hygiene, Milk

## Abstract

**Background:**

Milk is highly prone to contamination and can serve as an efficient vehicle for human transmission of foodborne pathogens, especially gram-negative bacteria, as these are widely distributed in the environment.

**Methods:**

This cross-sectional study of gram-negative staining bacterial contamination of milk meant for human consumption was carried out from October 2010 to May 2011 in Gondar town, Ethiopia. Milk samples were collected from critical control points, from production to consumption, that were hypothesized to be a source of potential contamination. Milk sampling points included smallholder’s milk producers, dairy co-operatives, a milk processing plant, and supermarkets. The hygienic procedures applied during milking, milk collection, transportation, pasteurization, and postpasteurization storage conditions at these specified critical control points were evaluated. Standard bacteriological cultivation and biochemical assays were used to isolate and identify bacterial pathogens in the milk samples.

**Results:**

The results of the current study showed that conditions for contamination of raw milk at different critical points were due to less hygienic practices in pre-milking udder preparation, sub-optimal hygiene of milk handlers, and poor sanitation practices associated with milking and storage equipments. Among all critical control points considered, transportation containers at milk collection centers and at processing plants were found to be the most heavily contaminated with gram-negative staining bacterial species. Overall, 54 different bacterial species were indentified, and *Escherichia coli* (29.6%), *Pseudomonas aeruginosa* (18.5%), and *Klebsiella pneumoniae* (16.7%), were the most commonly identified gram-negative staining bacterial pathogens. Of particular interest was that no gram-negative staining bacteria were isolated from pasteurized milk samples with varying shelf life.

**Conclusion:**

This study showed the presence of diverse pathogenic gram-negative staining bacterial species in raw milk that may be attributed to the sub-optimal sanitary conditions in the production and processing of milk in the Gondar town region. These results highlighted the need to maintain appropriate sanitary and hygienic measures at each critical point in order to safeguard consumers from foodborne pathogens. Further studies are recommended to identify additional critical control points, and to assess zoonotic risk factors to consumers.

## Background

Milk is largely made up of water, within which a wide range of nutrients including vitamins, proteins, fats and carbohydrates are suspended [1,2]. These rich nutritional contents and the production and processing procedures in commercial milk production render it susceptible to contamination by a host of pathogenic microbes that could cause diseases in humans. Therefore, milk is known to be an efficient vehicle for transmission of disease causing agents to humans [3].

Bacterial agents may contaminate milk at various stages of procurement, processing and distribution. Bacterial contamination could arise from the cow’s udder, barn, milk collection materials, various ingredients added to dairy products and dairy farm workers [4]. Gram-negative staining bacteria, which are the target of the present study, have been reported to contaminate milk as a consequence of milking cows affected by mastitis and from poorly sanitized utensils used during milking, transportation and storage processes [5]. These bacterial contaminants result in spoilage of milk and dairy products whenever there are appropriate growth conditions for these microorganisms to outgrow psychrotrophic bacteria [6].

Preventing the growth of contaminating bacteria in milk involves limiting contamination levels, cooling immediately after milking, and maintenance of cold storage temperatures. Limitation of bacteria primarily includes cleaning, sanitizing and drying cow’s teats and udder before milking and using sanitized milking equipments. Removal of residual solid milk from milk containers is critical in the control of psychrotrophic bacteria [7]. Pasteurization of milk has been practiced as the most effective method of reducing the risk of contamination and spreading of disease. Although pasteurized milk is expected to have a shelf life of 14 to 20 days, the shelf life of pasteurized milk stored at ambient temperature is dependent upon the efficiency of pasteurization process [8].

Bacteriological safety of milk continues to be a topic of concern in the dairy industry and public health communities. In general, in order to provide safe and healthy milk products, the Hazard Analysis and Critical Control Points (HACCP) system should be implemented starting from milk collection, through processing and storage. Microbial exposure assessments are critical components of the risk analysis [9-11].

In countries with poor milk production and marketing practices, one can expect a higher frequency of bacterial contamination, which poses health hazards as well as spoilage of large quantities of milk. In the current study area in particular, and in Ethiopia in general, there is a lack of information on the extent of raw and pasteurized milk contamination by gram-negative staining bacteria, despite the practice of door-to-door milk delivery by producers, and the prevailing habit of raw milk consumption in the country. In addition, to our knowledge, there has been no established milk quality control system. Therefore, the present study was initiated to provide base-line information on the quality of milk in order to identify critical control points, starting with production all the way to consumption by the public.

## Methods

### Study area

This study was carried out in the town of Gondar in the Northwestern part of Ethiopia. Gondar town is situated at longitude and altitude of 12.3-13.8°N, 35.3-35.7°E and 2200 meter above sea level, respectively. The annual mean minimum and maximum temperature of the area vary between 12.3-17.7 and 22–23°C, respectively. The town receives a bimodal rainfall, the average annual precipitation rate being 1000mm due to long and short rainy seasons [12].

### Study design and population

A cross-sectional study was conducted from October 2010 to May 2011 to detect and identify gram-negative staining bacterial contaminants in raw and pasteurized milk commonly consumed by the public. The study populations were lactating dairy cows from smallholder farms organized as cooperatives in and around Gondar town. These cooperatives were found in four administrative areas namely Tikil-Dingay, Arbaba, Gind-Metaya, and Shembekit. Each administrative area has its own milk collection center in which the milk from different members of the cooperatives tested for mastitis using Californian Mastitis Test and transferred to an aluminum container and transported to a processing plant. Each member of the cooperatives has one or more lactating cows.

### Ethical clearance

Ethical clearance was obtained from University of Gondar (Reference No R/P/O/04/436/07/08). Informed written consent was also obtained from all study participants and confidentiality was assured by the use of codes in records.

### Data collection and analysis

From the list of all dairy farm owners registered at Gondar town and its suburbs, 47 farms were selected using random sampling technique. A pre-tested and well-structured questionnaire was administered to lactating cow owners and workers to identify and evaluate potential risk factors that might influence the quality of milk. Risk factors considered in the current study were sanitary conditions of the barn, hygiene of cow’s udder and milk handlers, sanitation of containers, transportation and storage facilities, and shelf life of the pasteurized milk. Isolation and identification of bacterial species was carried out by conventional culture based techniques and biochemical assays. The data was entered into Microsoft Excel spread sheet and analyzed using Stata 11.

### Sampling and transportation

Milk samples were collected from points considered to be associated with contamination (critical control points: CCPs). The CCPs were the teat during milking (CCP-1), milking buckets at farm level (CCP-2), transport containers at milk collection centers (CCP-3), transportation containers up on arrival at the processing plant (CCP-4) and randomly selected pasteurized milk from pasteurization plant and at different time points from retailers (CCP-5). Overall, 107 raw and pasteurized milk samples were analyzed: of these, 92 were raw milk samples from CCP-1(n=34), CCP-2 (n=33), CCP-3 (n=12), and CCP-4 (n=13) and the remaining 15 were pasteurized milk samples from the processing plant and different retailer shops.

During sampling of raw milk directly from teats, the udder and teats were cleaned and dried before sampling; each teat end was scrubbed gently with cotton swabs moistened with 70% ethyl alcohol. The first 3–4 streams of milk were discarded, and approximately 10ml of milk was collected into sterile sampling bottles. The other raw and pasteurized milk samples were collected in the morning following standard procedures. Prior to sampling from milking buckets and transport containers, the milk was thoroughly mixed by shaking and 25ml of milk was transferred into a sterile screw capped bottle. Transportation of samples to the Microbiology Laboratory of Faculty of Veterinary Medicine, University of Gondar, was immediately conducted for further processing using ice packs following the standard safety procedures [13].

### Isolation and identification of gram-negative staining bacteria

Isolation and identification of gram-negative staining bacterial pathogens were determined following aseptic sampling techniques as described in Quinn et al. [14] and Barrow and Feltham [15]. Briefly: a loop full (0.01ml) of milk sample was streaked on 7% blood agar (Oxoide, Germany) and incubated aerobically at 37°C. The plates were checked for bacterial growth after 24, 48 and 72 hours to rule out slow growing micro-organisms and sub-cultured on blood agar at 37°C for 24 hours to get pure culture. A single colony from a pure culture was then subjected to Grams’ stain to observe morphological characteristics and transferred to brain heart infusion and MacConkey agar to be grown for further analysis.

Identification of bacteria to the species level was carried out based on their colony characteristics, Gram’s staining and morphological characteristics, growth on MacConkey agar, catalase, urease and oxidase tests, hydrogen sulfide (H_2_S) production, indole, methyl red (MR), and Voges-Proskauer (VP) reaction, citrate utilization, oxidation-fermentation test, motility, and different carbohydrate testes.

## Results

### Questionnaire survey

All farms included in this study had 1–6 cross-breed (Holstein-Friesian with indigenous) lactating cows with 50-75% exotic blood levels. About 8.5%, 59.6%, 17%, and 14.9% of the farmers cleaned their barn twice per day, every day, three times per week and once per week, respectively. Bedding condition of the barn in 66% of the cases was poor.

A quarter of dairy cows owners did not wash their cows’ udder before milking. Thirty-seven percent, 26%, 8.7%, and 2.4% of dairy farm owners washed their cow’s udders by warm tap water, cold river water, cold tap water, and warm river water, respectively. But none of the farmers used detergents, disinfectants or teat dips to clean teats and udders. In addition, none of them dried the udders. Most of the farmers (83%) cleaned their milking utensils every day but the remaining 17% of the farmers cleaned their milking utensils twice per day (4.3%) or once per two days (12.8%) using detergents. Hot water, which is indispensable for cleaning milking equipments, was used only by 44.7% of the farmers. Around 64% of the dairy farmers used traditional flavorings, such as smoke for the betterment of the smell of milking and transport equipments as shown in Table 1.

**Table 1:** Dairy farms hygienic practices

**Farmers practices**	**Performance**	**Number of farmers**	**Frequency(%)**
Barn cleaning	Twice per day	4	8.5%
	Once per day	28	59.58%
	Three times per week	8	17.03%
	Once per week	7	14.89%
Udder washing before milking	No washing at all	13	27.66%
	Using warm tape water	17	36.17%
	Using cold tape water	4	8.51%
	Using warm river water	1	2.13%
	Using cold river water	12	25.53%
Milking utensils cleaning	Twice per day	2	4.25%
	Every day	39	82.98%
	Every other day	6	12.77%
Milking utensils cleaning methods	Hot water	21	44.68%
	Cold water	26	55.52%
Use of flavoring	Traditional flavoring	30	63.83%
	No use of traditional flavoring	17	36.17%
Hand washing before milking	Yes	44	93.62%
	No	3	6.38%

Of the total dairy cow’s owners, 93.6% of them washed their hands before milking using detergent out of which 52.3% and 47.7% of them were using river and tap water, respectively. Milk collection workers at the sites of collection tests the quality and freshness of milk before individual farmers’ milk is added to the bulk tank milk.

### Isolation and identification of bacteria

Overall, a total of 54 bacterial isolates that were categorized under 9 different gram-negative staining bacteria species were identified from CCP-1, CCP-2, CCP-3 and CCP-4 (Table 2). From the 34 samples collected directly from cows’ udder (CCP-1), 32.4% yielded gram-negative staining bacteria. *Escherichia coli* (n=5), *Klebsiella pneumoniae* (n=4), *Enterobacter aerogenes* (n=1) and *Alcaligenes feacalis* (n=1) were the identified isolates. At this sampling point no mixed bacterial species contamination was observed.

**Table 2:** Frequency distribution of bacteria isolates from different critical control points

**Bacterial species**	**CCP-1**	**CCP-2**	**CCP-3**	**CCP-4**	**Total**
*Escherichia coli*	5(45.46)	4(30.77)	4(26.66)	3(20.00)	16(29.63)
*Pseudomonas aeruginosa*	0	3(23.08)	4(26.66)	3(20.00)	10(18.52)
*Klebsiella pneumoniae*	4(36.36)	2(15.39)	3(20.00)	0	9(16.67)
*Enterobacter aerogenes*	1(9.09)	1(7.69)	1(6.67)	1(6.67)	4(7.41)
*Citrobacter freundi*	0	1(7.69)	1(6.67)	2(13.33)	4(7.41)
*Proteus mirabilis*	0	1(7.69)	1(6.67)	1(6.67)	3(5.56)
*Proteus vulgaris*	0	0	0	2(13.33)	2(3.70)
*Alcaligenes feacalis*	1(9.09)	1(7.69)	1(6.67)	2(13.33)	5(9.26)
*Acinetobacter calcoaceticus*	0	0	0	1(6.67)	1(1.85)
**Total**	**11**(**100**%)	**13**(**100**%)	**15**(**100**%)	**15**(**100**%)	**54**(**100**%)

Numbers in parenthesis indicate percentages.

Among the 33 milk samples collected from the milking bucket, 39.4% were culture positives for gram-negative staining bacteria. Bacterial species identified from this critical control point (CCP-2) included *Escherichia coli* (n=4), *Pseudomonas aeruginosa* (n=3), *Klebsiella pneumoniae* (n=2), *Enterobacter aerogenes* (n=1), *Alcaligenes feacalis* (n=1), *Proteus mirabilis* (n=1) and *Citrobacter freundi* (n=1). Only a single bacterial species were identified from this CCP.

All the 12 milk samples collected from storage containers at CCP-3 before transportation to the main pool were culture positives. Three samples had more than one bacterial species. Types of isolates identified in this critical point were similar with those identified at CCP-2 except a slight increase in *Pseudomonas aeruginosa* and *Klebsiella pneumoniae*. Similarly, all of the milk samples collected at CCP-4 showed gram-negative staining bacterial contamination. Two samples had mixed bacterial contamination; *Proteus vulgari*s and *Acinetobacter calcoaceticus*. None of the pasteurized milk samples taken from the processing plant and various supermarkets of different shelf life yielded gram-negative staining bacteria.

## Discussion

The result of the study questionnaire indicated that most of the sampled dairy cows were reared under unclean environmental conditions and poor udder preparation. Therefore, it is likely that raw milk might be contaminated from manure, soiled bedding and soil [16]. In addition, water used for cleaning the milking equipment and washing hands has been associated with potential source of gram-negative staining bacterial contamination in bulk tank milk [17] which might hold true in this study. Robinson [13] suggested that prior to using detergents, it is essential that the equipment be washed with cold water to remove as much previous milk and dirt as possible followed by washing with warm water to remove fatty deposits. Afterwards, the equipment has to be washed again with warm water and stored in a clean, dry and dust free area. As this research result reveals, this was not practiced by 44.7% of the dairy farm owners.

All of the bacteria identified from the milk samples collected directly from the cows’ udder (CCP-1) were coliform bacteria including, *Escherichia coli*, *Klebsiella pneumoniae*, *Alcaligenes feacalis* and Enterobacter earogenes, with frequency of 47.1%, 26.5%, 14.7%, and 11.8%, respectively. Higher isolation frequencies, especially for *Escherichia coli* and *Klebsiella pneumoniae* was observed in the current study as compared to similar studies performed to assess bacteriological quality of raw milk in Ethiopia [18-20]. This might be due to poor and unhygienic bedding condition in the majority of farms and absence of teat dipping and disinfection practices in the current study. These practices have been known as critical components of mastitis prevention and control program in dairy herds [21].

Bacteria cultivated from farmers bucket at farm level were also dominated by coliform bacteria which accounted 69.2% of the total isolates. *Pseudomonas aeruginosa*, *Citrobacter freundi*, and *Proteus mirabilis* were identified species at this critical control point, in addition to those identified at the previous critical point (CCP-1). Other studies have shown that milk from the bucket at the farm level could be contaminated with gram-negative staining bacteria present on teats, teat canal, udder surface, mastitis udder, residents milking system, and contaminated water used to clean the milking systems [22]. The apparent dominance of coliform bacteria in this study might be due to the fact that dairy farm owners did not use detergents and disinfectants to wash cows’ udder, which could have significantly reduced the level of coliform and some non-coliform bacteria. Karakök [23] reported a similar observation in that use of detergents could reduce contamination of milk by bacteria. A relatively higher number of *Pseudomonas aerogenosa* and *Escherichia coli* isolates were detected in the present study than in the reports of Alehegne [19], Formm and Boor [24], and Godefay and Molla [2]. It is likely that the psychrotrophic *Pseudomonas aerogenosa* was originally derived from soil and water. Hence, this organism could gain access into bucket through untreated water used to clean the milking systems or improper udder preparation before milking as well as direct contact with fecal material during milking. The organism has previously generally been mostly isolated from raw milk of bulk thank milk [25].

Bacterial isolates from storage container before transportation at milk collection center were similar to isolates identified at the preceding critical point (CCP-2). This finding was similar to those reported by Bashir and Usman [26]. Similar to our findings at this particular critical point, Khan et al. [27] reported that the dominant gram-negative staining bacteria associated with raw milk samples from bulk tank milk were *Escherichia coli* followed by *Pseudomonas aeruginosa*. This might result from poor milking hygiene, unnecessary mixing, transfer of milk from can to can and long milk collection rounds coupled with high ambient temperature.

The detection of additional bacterial species such as *Proteus vulgaris* and *Acinetobacter calcoaceticus* which were not isolated in the previous critical control points as well as the increased frequency of isolates like *Alcaligenes feacalis* and *Citrobacter freundi* from CCP-4 might be attributed to higher environmental contamination during transportation and/or contamination during waiting along the roadside. Milk in this situation could be exposed to contamination by vehicle dust, since the road is made of gravel. This result is lower in frequency compared with a report by Lues et al. [28] from South Africa.

All pasteurized and packed milk samples taken from various supermarkets and restaurants at different shelf life were culture negative for gram-negative staining bacteria. This may be explained by the effectiveness of pasteurization at the processing plant that minimized the chance of postpasteurization contamination. This might also be attributed to a lower level contamination of the milk along the path from cow to the pasteurization plant. As Ashenafi and Beyene [29] indicated, microorganisms could survive pasteurization temperature if there is high level of contamination of raw milk. Therefore, keeping such pasteurized milk at room temperature for several hours in retail shops or at home may lead to early spoilage of milk. A high contamination of raw milk implies a higher chance for micro-organisms to survive pasteurization. Psychrotrophic bacteria are important here because many of them can produce extracellular thermostable proteolytic and lipolytic enzymes which can survive pasteurization thus affecting the shelf life and quality of milk and milk products during storage [2].

Overall, 61.1% of the bacterial isolates identified at this particular study area belonged to coliform bacteria. Among which, *Escherichia coli* (30.2%), *Pseudomonas aeruginosa* (18.9%) and *Klebsiella pneumoniae* (17%), were the first, second and third dominant bacterial isolates identified from milk consumed in and around Gondar town, respectively. Most of the bacteria identified here are indicators of poor sanitary and hygienic condition of the farms and transportation system which is similar with previous report by Parekh and Subhash, 2008 [30]. The high presence of *Escherichia coli* in the milk samples imply that faecal contamination could have occurred and subclinically ill cows might have served as the causes of the microbial contamination. Traditional practices are likely to contribute to the contamination of the milk and proliferation of the micro-organisms. The implication is that there is high risk of acquiring foodborne diseases since 36% of the residents have the habit of consuming raw or unpasteurized milk. It is therefore essential to implement control measures at each critical control points identified in our investigation.

## Conclusions

Milk is an excellent nutrient medium for the growth of pathogenic bacteria that contaminate milk from various sources at different critical control points. The result of bacteriological assessment of raw milk contaminants at different critical control points in this study showed that diverse environmental bacteria could contaminate milk. The presence of high coliform isolates from critical control point one may be attributed to high prevalence of subclinical coliform mastitis, unclean dairy houses, improper milking procedure and udder preparation, negligence on post-milking teat dipping and lack of herd health management. A high numbers of coliform and non-coliform isolates form critical control point two, three, and four may be due to the use of contaminated water, absence of detergents and/or disinfectants to wash udders and milk equipment and environmental contamination from dusty road during transportation. It is recommended that educating the dairy farm owners about microbial safety procedures from the point of udder sanitary processes to the sanitary practices at collection centers, should be mandatory to improve the hygienic quality of milk and minimize contamination. Furthermore, clean and easily accessible water should be provided for dairy farm owners who currently do not have such access. Provision of cooling systems at milk collection sites and fast and dust proof transportation systems will also play key roles in reducing contamination of the milk. Finally, hazard analysis critical control point system implementation and control measures from farm to postpasteurization milk handling should be undertaken in order to minimize food borne diseases in this area.

## Competing interests

The authors declare that there is no financial or non-financial competing interest from any body or institute. We also want to assure that we did not receive any technical assistant in developing the research concept or preparation of the manuscript.

## Authors' contributions

LG carried out the conception of the research concept and design the methodology, data analysis and interpretation and preparation of the manuscript for publication. AB carried out the laboratory work, data collection and revision of the manuscript. DM critically revised the proposal, designed the methodology, and reviewed the manuscript. GTs carried out the laboratory work and reviewed the manuscript. All authors read and approved the final manuscript.

## Pre-publication history

The pre-publication history for this paper can be accessed here:

http://www.biomedcentral.com/1471-2458/12/950/prepub
